# Expression Characteristics of SERINC1 and TDO2 in Diabetic Kidney Disease: Historical Candidate Reappraisal, Cross-Cohort Evaluation, Single-Cell Analysis, and a Mouse Model Study

**DOI:** 10.3390/ijms27219418

**Published:** 2026-10-22

**Authors:** Xiaofei Cui, Yijun Ren, Guohua Ding, Sen Chen

**Affiliations:** 1 Division of Nephrology, Renmin Hospital of Wuhan University, Wuhan 430060, China; 2 Nephrology and Urology Research Institute, Wuhan University, Wuhan 430060, China

**Keywords:** diabetic kidney disease, SERINC1, TDO2, single-cell RNA sequencing, glomerular transcriptomics, model validation

## Abstract

Evaluating candidate molecules in diabetic kidney disease (DKD) requires evidence for both the reproducibility of expression changes and their cellular origin. Using the historically fixed SERINC1/TDO2 panel, we reappraised how these candidates were derived, evaluated both genes in human glomerular transcriptomes and kidney-cortex single-cell data, and examined their relationship with renal injury in a mouse model. The historical WGCNA, LASSO, and SVM-RFE-labelled frozen lists intersected at SERINC1 and TDO2, although the legacy RFE implementation could not be confirmed. In the current 83-sample analysis, 584 differentially expressed genes were detected; intersecting the cross-cohort direction-consistent differentially expressed genes with the disease-associated WGCNA module retained 26 candidates that did not include the two targets, whereas machine learning applied to a topology-derived subset of these candidates identified DUSP1. TDO2 was increased in both cohorts (Hedges’ g = 2.07 and 0.714; the latter Holm-adjusted *p* = 0.0182), whereas the SERINC1 reduction was supported only in the discovery cohort (g = −1.90) and not in GSE96804 (g = −0.252; Holm-adjusted *p* = 0.348). Single-cell analysis showed widespread SERINC1 distribution without donor-level significance after Benjamini–Hochberg correction, and TDO2 was detected in only 50 of 37,331 cells. In streptozotocin-induced mice, SERINC1 decreased and TDO2 increased alongside renal pathology and podocyte ultrastructural changes. Both genes remain candidates for further study, but clinical diagnostic value and shared pathogenic mechanisms are not yet established.

## 1. Introduction

Diabetic kidney disease (DKD) is a major cause of chronic kidney disease and end-stage renal disease [1,2]. The molecular features of glomerular injury may reflect the disease process, but they may also be shaped by tissue composition, disease stage, and clinical context. Candidate molecules should therefore be judged by whether expression changes can be reproduced across different sample sources, rather than by a difference in a single cohort or by the discriminatory capacity of a model.

Podocytes maintain the glomerular filtration barrier, and foot-process effacement, basement-membrane thickening, and mitochondrial changes provide the pathological context of glomerular injury, which can progress to podocyte loss through regulated cell death [3,4,5]. Bulk tissue transcriptomes identify disease-associated genes and co-expression modules, but their signal also contains the expression of many cell types and shifts in their abundance [6,7]. Candidates emerging from machine learning screening must still be tested against independent data; a relatively high AUC in the development data cannot substitute for cross-cohort evaluation.

Single-cell RNA sequencing (scRNA-seq) can reveal the cellular origin of tissue expression signals, but low-abundance transcripts and few donors constrain between-group inference [8,9]. Combining single-cell, tissue, and animal results requires answering three questions separately: whether the expression direction is reproduced, in which cells the signal resides, and whether gene changes accompany renal injury. The different levels may agree or diverge depending on sampling and detection conditions.

SERINC1 (TDE2) was initially linked to serine-dependent membrane lipid synthesis through the Serinc family, although later loss-of-function models suggest context-dependent function [10,11]. TDO2 encodes tryptophan 2,3-dioxygenase, which catalyzes the initial reaction of the kynurenine pathway; recent kidney studies report cellular and metabolite changes in this pathway, but these do not establish a podocyte role for TDO2 [12,13]. The biology of the two genes frames our research question, yet the reproducibility and cellular origin of their expression in DKD remain to be assessed directly.

**Figure 1:**
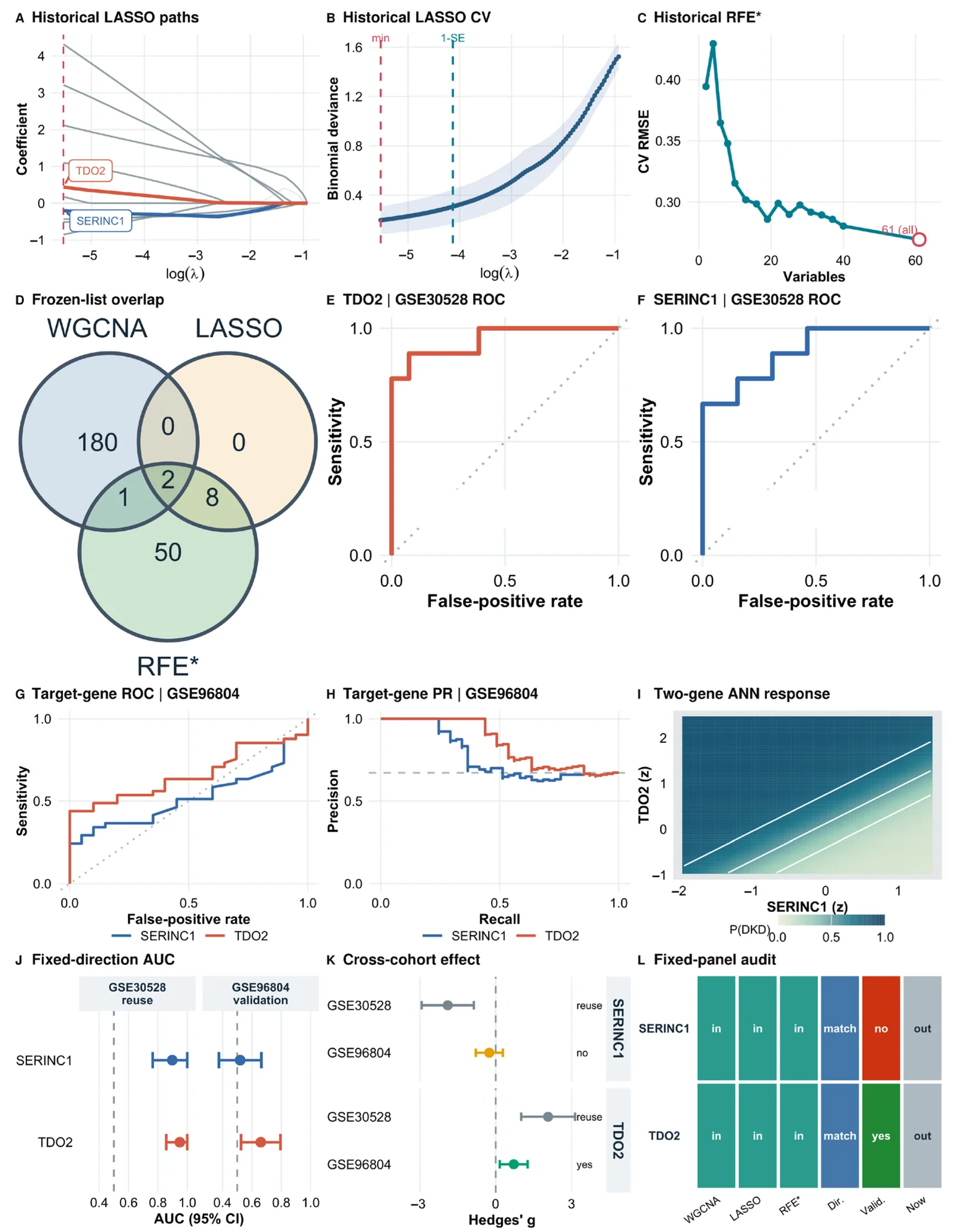
Historical candidate origin of SERINC1 and TDO2 and overview of the current evidence. (**A**) LASSO coefficient paths for the historical GSE30528 data, with SERINC1 and TDO2 highlighted. (**B**) Binomial deviance of the 10-fold cross-validation (shading, ±1 estimated SE; dashed lines, λmin and λ1SE). (**C**) RFE error curve restored from the historical PDF (61 = all input variables retained). (**D**) Intersection of the frozen WGCNA green module (183 genes), LASSO (10), and historical RFE (61) lists, yielding SERINC1 and TDO2. (**E**,**F**) Fixed-direction ROCs with DeLong 95% CIs in GSE30528 (reuse of discovery data). (**G**,**H**) Fixed-direction ROCs and precision–recall curves in GSE96804 (dashed line, DKD proportion 0.672); SERINC1 blue, TDO2 orange-red. (**I**) Locked two-gene ANN response surface (contours, predicted P(DKD) 0.25/0.50/0.75; grey, outside the development range). (**J**) Fixed-direction AUCs and 95% CIs (dashed line, AUC = 0.5). (**K**) Hedges’ g and 95% CIs (dashed line, zero effect). (**L**) Historical inclusion, direction consistency, candidate-level confirmation, and current screening status. RFE* retains the historical label and is not equivalent to the current linear SVM-RFE.

This study followed the historically fixed SERINC1/TDO2 panel. We first reappraised the frozen candidate lists and the historical LASSO and RFE records against the current conditional screening (Figure 1); we then evaluated expression associations and model performance in two glomerular cohorts, described cellular distribution in kidney-cortex single-cell data from an independent source, and finally combined these with pathological and expression observations in streptozotocin (STZ) mice. Pathway, regulatory network, colocalization, and structural analyses were used to propose testable explanations. The focus was the level of support each gene received across these layers, rather than redefining them as new discoveries from the current genome-wide screening.

## 2. Results

### 2.1. Historical Candidate Reappraisal and Overview of Cross-Cohort Evidence for the Fixed Two-Gene Panel

Figure 1 summarizes candidate origin and current evidence. The historical LASSO on GSE30528 matched the frozen list (10 genes; Figure 1A,B). The curve originally labelled SVM-RFE reached its lowest error at 61 variables, with no variable reduction; given the legacy script’s problems, it is shown only as a historical RFE record (RFE*; Figure 1C). The intersection of the WGCNA green module (183 genes), LASSO (10), and RFE (61) lists still yielded SERINC1 and TDO2 (Figure 1D).

With direction fixed, GSE30528 AUCs were 0.949 (95% CI 0.856–1.000) for TDO2 and 0.897 (0.764–1.000) for SERINC1 (Figure 1E,F; reuse of discovery data), and 0.660 (0.526–0.794) and 0.520 (0.376–0.664) in GSE96804 (Figure 1J). TDO2 increased in both cohorts; the SERINC1 reduction in GSE96804 was not confirmed (Figure 1K). GSE96804 AP was 0.772 (SERINC1) and 0.849 (TDO2) against a DKD proportion of 0.672 (Figure 1H); fixed-direction ROCs are in Figure 1G. The locked ANN response surface (Figure 1I) describes model output without demonstrating interaction or replacing cross-cohort evaluation. Neither gene entered the six-gene set; Figure 1L distinguishes historical inclusion, direction consistency, candidate-level confirmation, and screening status.

### 2.2. Differential Expression and Co-Expression Context of the Glomerular Transcriptome

**Figure 2:**
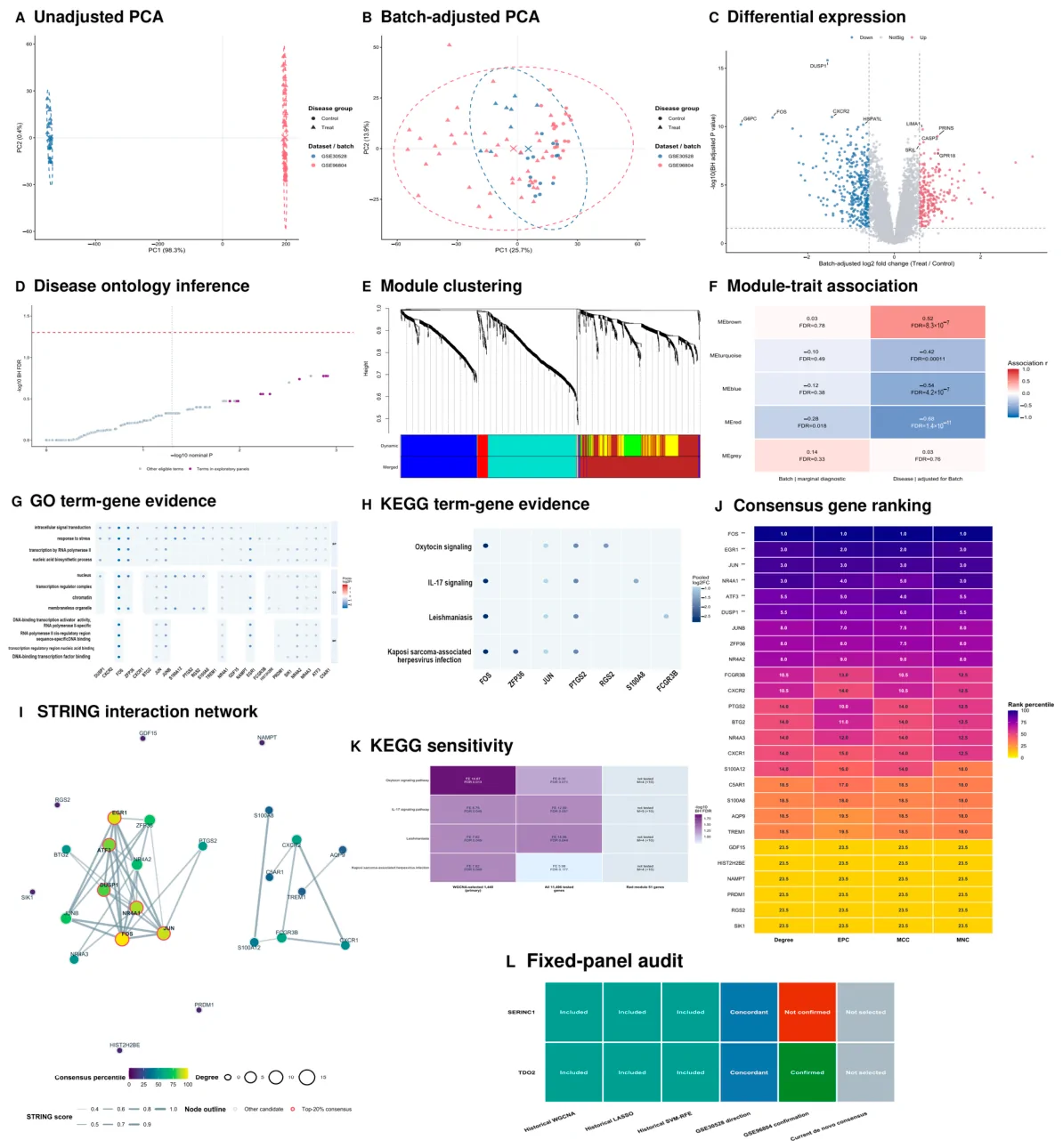
Differential expression, co-expression context, and candidate origin. (**A**,**B**) PCA before and after ComBat correction (color/shape, source, and disease group). The dashed circles in (**B**) represent the grouping boundaries (confidence ellipses) for different sample groups. (**C**) Differential expression volcano plot. The dashed lines in (**C**) represent the reference boundaries for the sample distribution. (**D**) Nominal P versus BH-adjusted FDR in the Disease Ontology analysis (dashed line, FDR = 0.05). (**E**) WGCNA dendrogram and module merging. (**F**) Module eigengene associations with disease and batch. (**G**,**H**) GO and KEGG candidate member matrices. (**I**) STRING network (confidence 0.700, isolated nodes retained). (**J**) Percentile topological rankings of Degree, EPC, MCC, and MNC, and the asterisks (**) indicate statistical significance (*p* < 0.01). (**K**) Sensitivity of significant KEGG terms across backgrounds. (**L**) Origin and evidence status of the fixed two-gene panel.

The 83 samples showed cohort-related structure; ComBat reduced separation, and disease estimates stayed highly correlated (r = 0.988; Figure 2A,B). Of 11,406 genes, 584 were DEGs (235 up, 349 down); 477 (81.68%) had concordant directions, but the cohort × group interaction passed FDR for 270, indicating residual heterogeneity (Figure 2C). WGCNA merged six modules into four non-grey; the red module (51 genes) showed the strongest batch-adjusted DKD association (partial correlation −0.677, FDR 1.43 × 10^−11^; Figure 2E,F). Intersecting concordant DEGs with the red module retained 26 candidates, all decreased.

With the WGCNA background, GO yielded 117 significant terms (83 BP, 12 CC, and 22 MF); significant KEGG terms were oxytocin signaling, IL-17 signaling, leishmaniasis, and Kaposi sarcoma-associated herpesvirus infection (sharing JUN, FOS, and PTGS2; Figure 2G,H). Using all genes reduced significant KEGG terms to one (Figure 2K), and no Disease Ontology term reached significance (lowest FDR 0.168; Figure 2D). The STRING network had 26 nodes, 41 edges, and 6 isolates, led by FOS, EGR1, JUN, NR4A1, ATF3, and DUSP1 (Figure 2I,J). SERINC1 and TDO2 were retained as the historical panel (Figure 2L).

### 2.3. Cross-Cohort Expression Support for TDO2 and Transfer Performance of the Two-Gene Model

**Figure 3:**
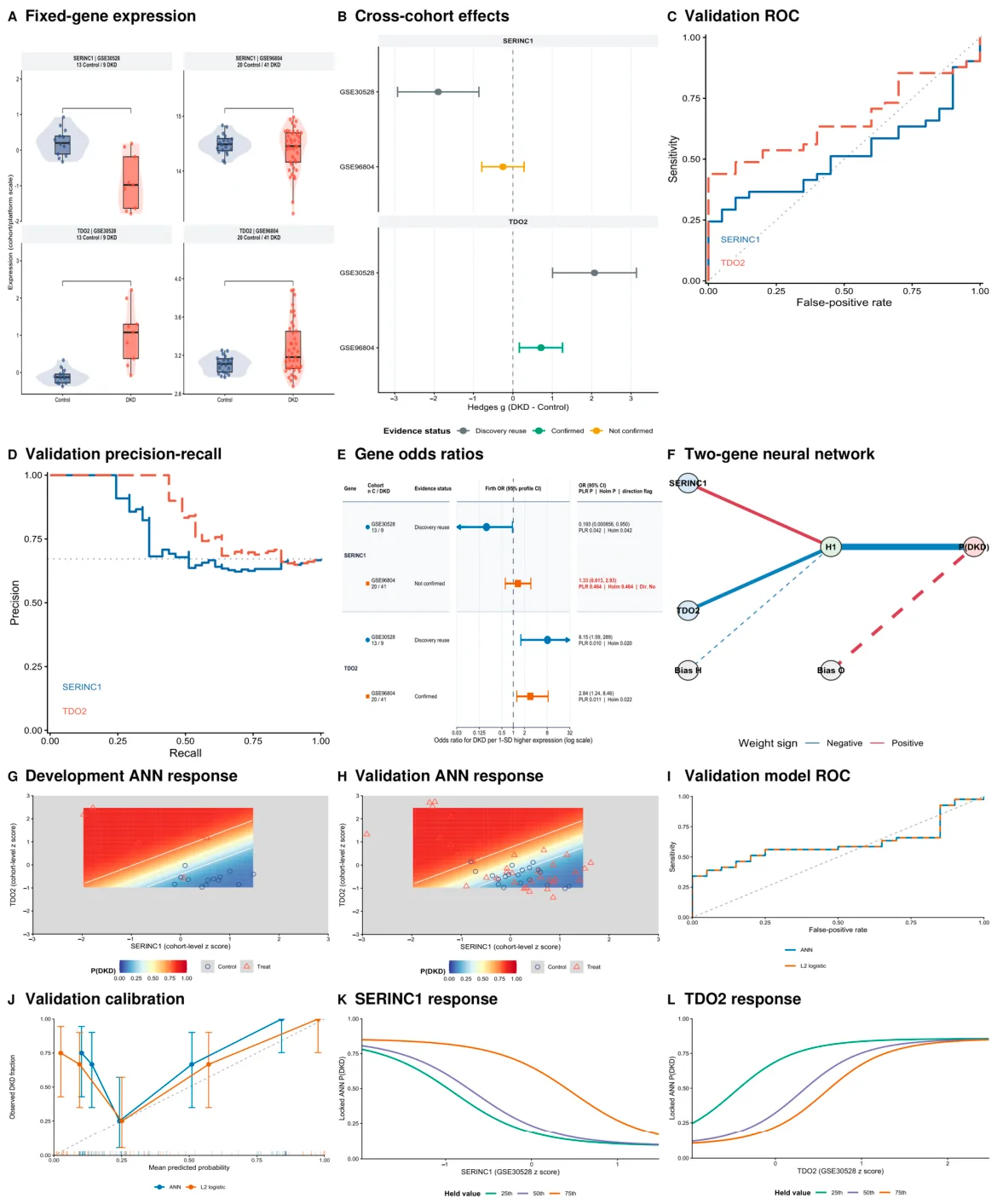
Expression associations and model evaluation. (**A**) Expression distributions in the two cohorts. Blue represents the Control group and red represents the DKD group. (**B**) Hedges’ g and 95% CIs. The vertical dashed line indicates the null hypothesis baseline of 0. (**C**,**D**) Fixed-direction ROC and precision–recall curves in GSE96804 (reference line, DKD proportion 0.672). Different colors indicate different genes (SERINC1 in blue and TDO2 in red). The dashed lines represent the random chance diagonal in (**C**) and the reference threshold of 0.7 in (**D**). (**E**) Firth logistic regression: ORs and profile-likelihood 95% CIs per within-cohort SD. The vertical dashed line indicates the reference threshold of 0.7. (**F**) Locked ANN 2-1-1 structure and weights. Different colors indicate different genes, and dashed lines represent the negative weight sign). (**G**,**H**) ANN response surface and contours (grey, outside the development range). (**I**) ROC comparison of the ANN and L2 logistic model in GSE96804. (**J**) Group-wise calibration. (**K**,**L**) Single-gene response curves with the other gene at development-cohort quartiles.

In GSE30528, SERINC1 decreased and TDO2 increased (Hedges’ g = −1.90, 95% CI −2.93 to −0.86; and 2.07, 1.01–3.14; Holm-adjusted *p* ≈ 0.00035 each), a reappraisal of discovery data. GSE96804 supported TDO2 (g = 0.714, 0.164–1.263; *p* = 0.0182) but not SERINC1 reduction (g = −0.252, −0.789 to 0.284; *p* = 0.348; Figure 3A,B). Fixed-direction AUCs were 0.897 and 0.949 (GSE30528) and 0.520 (0.376–0.664) and 0.660 (0.526–0.794) (GSE96804); AP was 0.772 and 0.849 against a DKD proportion of 0.672, so AP must be interpreted with reference to sample composition (Figure 3C,D). In the mutually adjusted Firth model, the TDO2 OR was 2.845 per SD (1.238–8.455; *p* = 0.022); the SERINC1 OR was 1.330 (0.613–2.926; *p* = 0.464), opposite to the historical hypothesis (Figure 3E).

The final ANN (2-1-1, weight decay 0.1) locked a threshold of 0.6533 (Figure 3F). Development out-of-fold AUC was 0.889 (Brier 0.082); in GSE96804, AUC 0.609 (0.466–0.751), AP 0.824, Brier 0.316, sensitivity 36.6%, specificity 95.0%, calibration intercept 1.760, and slope 0.428. The L2-penalised logistic model matched the AUC (no ANN advantage; Figure 3I,J). Response surfaces and single-gene curves show model response, not intervention effects (Figure 3G,H,K,L).

### 2.4. Kidney-Cortex Single-Cell Atlas and Cell Annotation

**Figure 4:**
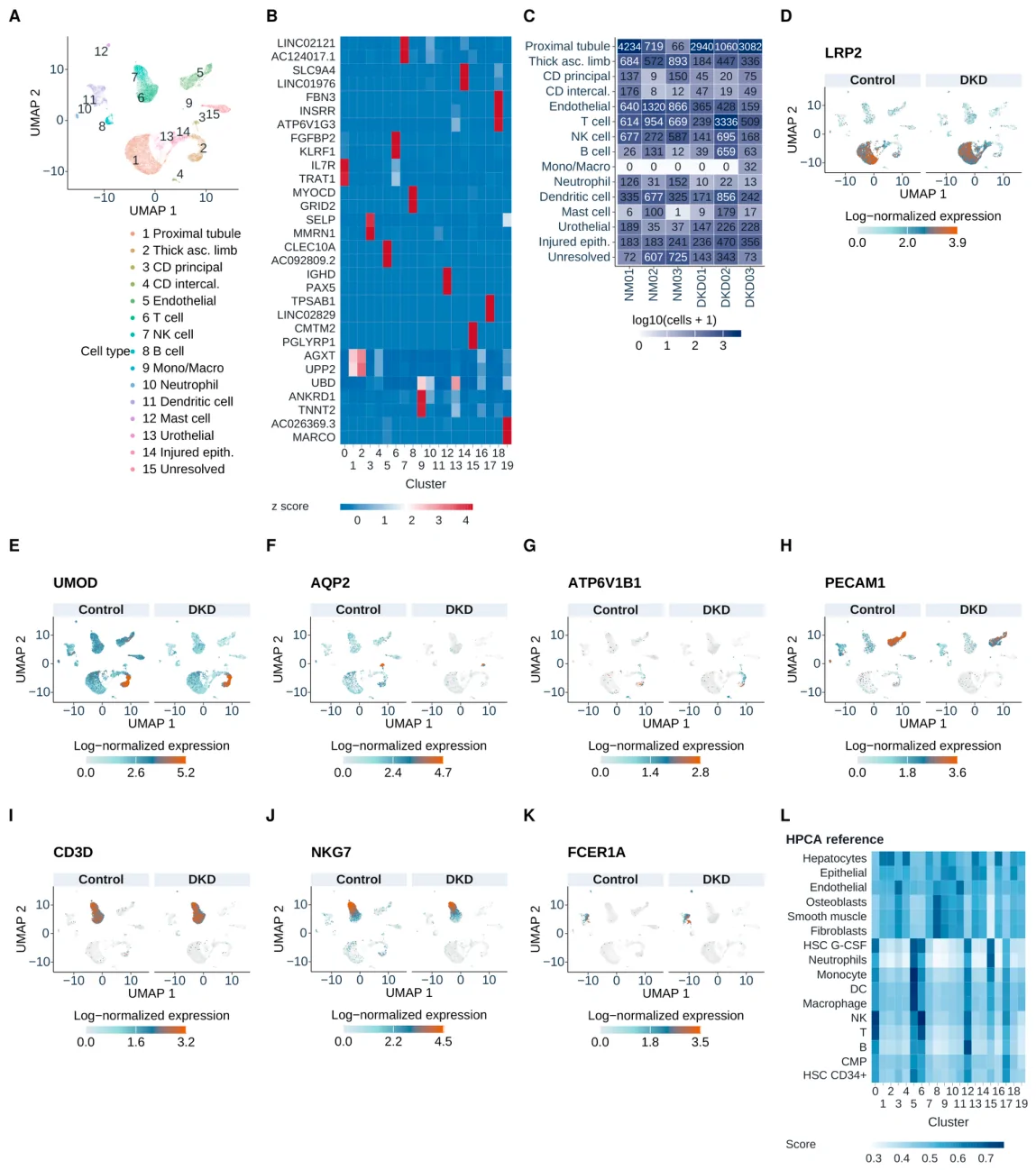
Kidney-cortex single-cell atlas and annotation. (**A**) UMAP of 37,331 cells (numbers, 14 annotated cell types, and Unresolved). (**B**) Standardised expression of 30 marker genes across 20 clusters (axis 0–19). (**C**) Cell counts per donor and type (colour, log10(count + 1)). (**D**–**K**) Expression of LRP2, UMOD, AQP2, ATP6V1B1, PECAM1, CD3D, NKG7, and FCER1A. (**L**) SingleR cluster-level scores (Human Primary Cell Atlas). The UMAP reflects expression similarity, not anatomical position; SingleR scores were auxiliary.

EmptyDrops identified 131,681 droplets; quality control retained 40,885 cells; and scDblFinder removed 3554 doublets, leaving 37,331 cells (8099; 5618; 4736; 4716; 8760; and 5402 per donor). Twenty clusters were annotated into fourteen cell types plus Unresolved (Figure 4A).

Thirty marker genes are shown in the main figure; eight cell types met ≥20 cells per donor in all donors (Figure 4B,C). LRP2, UMOD, AQP2, and ATP6V1B1 supported proximal tubule, thick ascending limb, principal cells, and intercalated cells; PECAM1, CD3D, NKG7, and FCER1A supported endothelial cells, T, NK, and dendritic cells (Figure 4D–K). SingleR scores were auxiliary (Figure 4L). The Unresolved class (1963 cells) included a candidate monocyte/macrophage cluster of 32 cells (all DKD03), insufficient for a DKD-specific population, and no comparable podocyte population; the atlas provides background without podocyte-specific validation.

### 2.5. Cellular Distribution of SERINC1 and the Limit Imposed by Low TDO2 Detection

**Figure 5:**
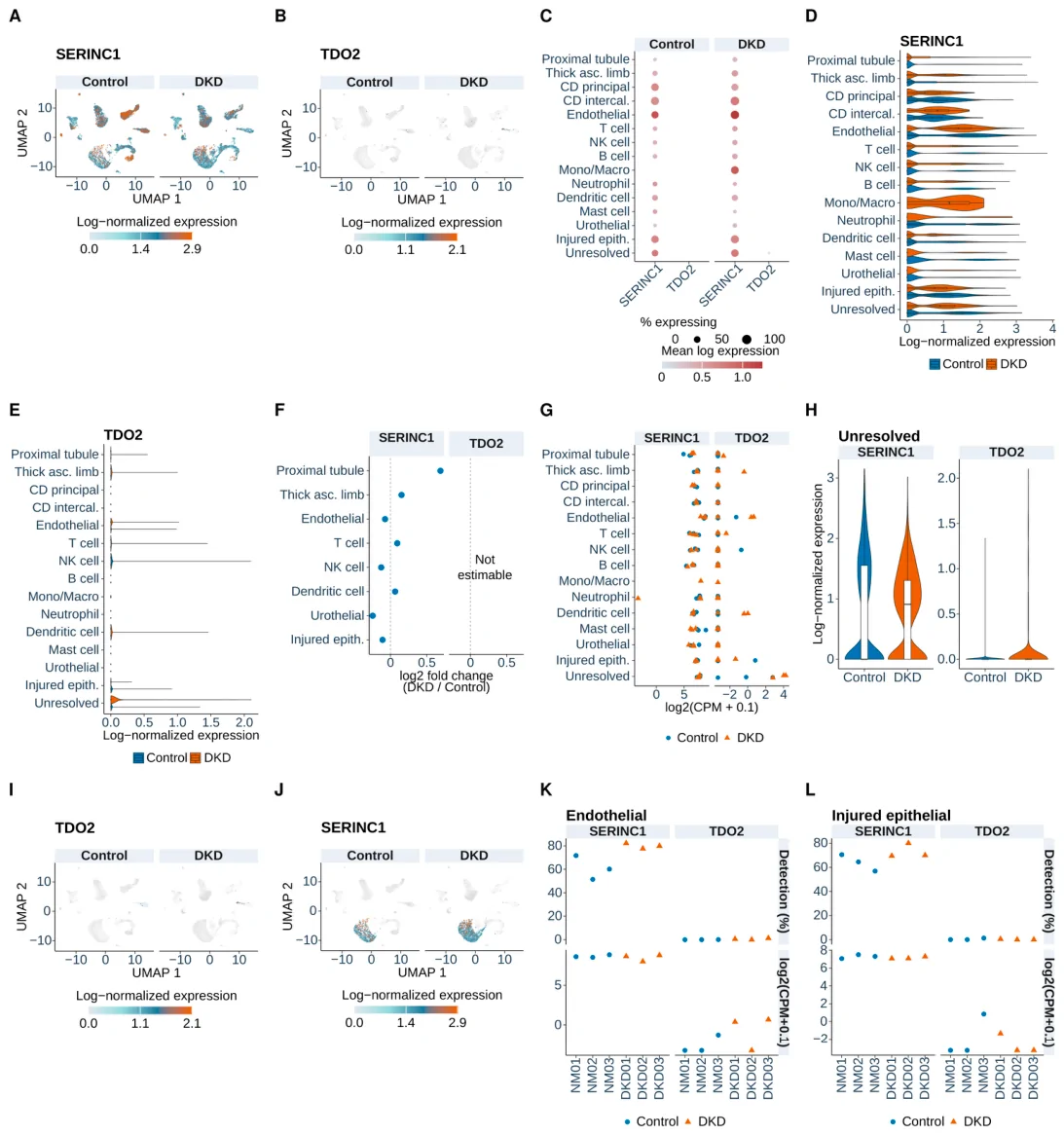
Cellular distribution and donor-level comparison. (**A**,**B**) UMAP expression of SERINC1 and TDO2 (shared scale between groups). (**C**) Detection proportion and mean log-normalized expression by cell type. (**D**,**E**) Expression distributions with zero-count cells retained. (**F**) Donor-level pseudobulk effects in eight cell types (SERINC1: BH-adjusted FDR = 1 across 28 hypotheses; TDO2: not estimable). (**G**) Donor-level log2(CPM + 0.1). (**H**,**I**) Unresolved-class expression and TDO2 UMAP. (**J**) SERINC1 UMAP (proximal tubule background). (**K**,**L**) Endothelial and injured epithelial detection proportions and log2(CPM + 0.1). Blue, control; orange, DKD. The donor is the replicate unit.

SERINC1 was detected in 13,398 cells and TDO2 in 50 of 37,331 cells; feature plots, detection proportions, and distributions showed broad SERINC1 but sparse TDO2 signal (cell-level, not donor-level; Figure 5A–E). All eight qualifying cell types gave estimable SERINC1 pseudobulk results, but the FDR was 1 across the 28 fixed hypotheses; the proximal tubule log2FC was 0.688 (nominal *p* = 0.0495; genome-wide FDR 0.551; and family FDR 1), opposite to the expected reduction (Figure 5F,G,J). TDO2 failed filterByExpr in all cell types. Of 50 TDO2-positive cells, 35 were in Unresolved (2/1404 control vs. 33/559 DKD), dependent on donor composition and annotation uncertainty (Figure 5H,I). SERINC1 log2FC was approximately −0.076 (endothelial) and −0.110 (injured epithelial), non-significant after correction (Figure 5F,K,L). These add distributional information without reproducing a general SERINC1 reduction; sparse TDO2 leaves its glomerular correspondence unresolved.

### 2.6. Exploratory Pathway Associations and Supplementary Colocalization

**Figure 6:**
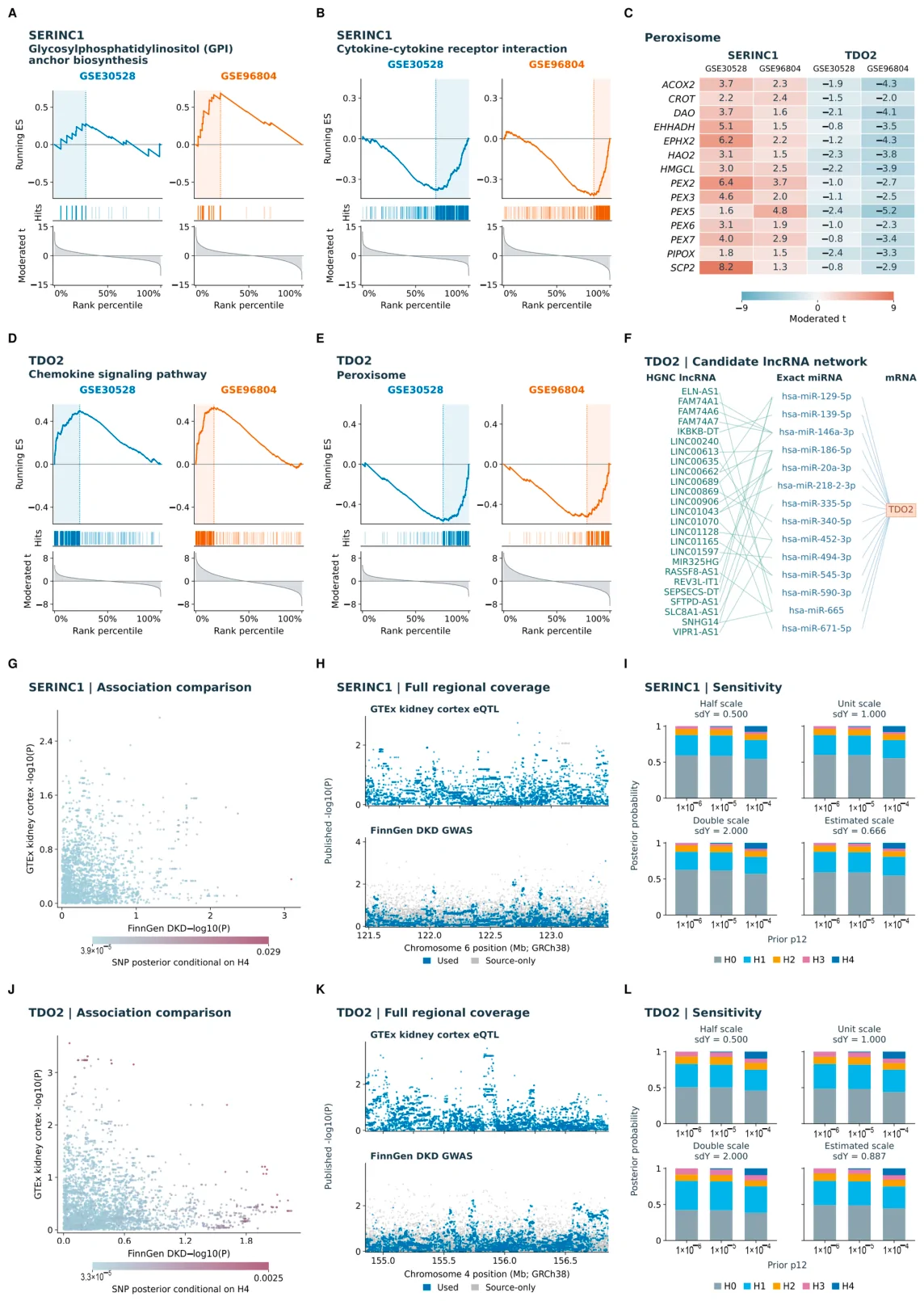
Pathway associations, candidate network, and colocalization. (**A**,**B**) SERINC1 rankings with GPI-anchor biosynthesis and cytokine–receptor interaction. (**C**) Fourteen-gene peroxisomal leading-edge intersection (raw moderated t). (**D**,**E**) TDO2 rankings with chemokine signaling and peroxisomes. (**A**,**B**,**D**,**E**) GSE30528 and GSE96804 side by side (NES, fgsea ranking metric). (**F**) TDO2 lncRNA-miRNA-mRNA network. (**G**,**J**) eQTL-GWAS statistics for shared variants (color, posterior probability conditional on H4). (**H**,**K**) Regional association plots. (**I**,**L**) H0–H4 posteriors under varying scales and priors. Reference H4 = 0.00859 (SERINC1) and 0.01026 (TDO2).

Consistent between-cohort ranking directions included SERINC1 positively with GPI-anchor biosynthesis and negatively with cytokine–receptor interaction, and TDO2 positively with chemokine signaling and negatively with the peroxisomal set (Figure 6A,B,D,E). The TDO2 peroxisomal set had fgsea NES −2.358 and −2.252 but CAMERA FDR 0.502 and 0.693; no pathway reached CAMERA FDR < 0.05, so negative ranking does not imply TDO2-driven peroxisomal suppression. The peroxisomal leading-edge intersection comprised 14 genes (ACOX2, EHHADH, SCP2, and PEX members), and ranked positive with SERINC1 and negative with TDO2 in the raw moderated t heat map—descriptive, as an already selected common leading edge (Figure 6C). The TDO2 network retained 25 lncRNAs, 14 miRNAs, and 1 target (40 nodes, 43 edges, and 29 paths), unverified by co-expression or intervention (Figure 6F).

Colocalization matched 4859 (SERINC1) and 6089 (TDO2) shared variants; H4 posteriors were 0.00859 and 0.01026 (0.86% and 1.03%), with no strong shared-causal support (Figure 6G–L). Shared variants covered ~95.9%/96.3% of eQTL but only ~33.9%/37.7% of GWAS variants, omitting both GWAS leading variants. Given the small eQTL sample (73), weak signals, and prior/scale sensitivity, low H4 was not interpreted as excluding a genetic association.

### 2.7. Candidate Docking Poses of SERINC1 and TDO2

**Figure 7:**
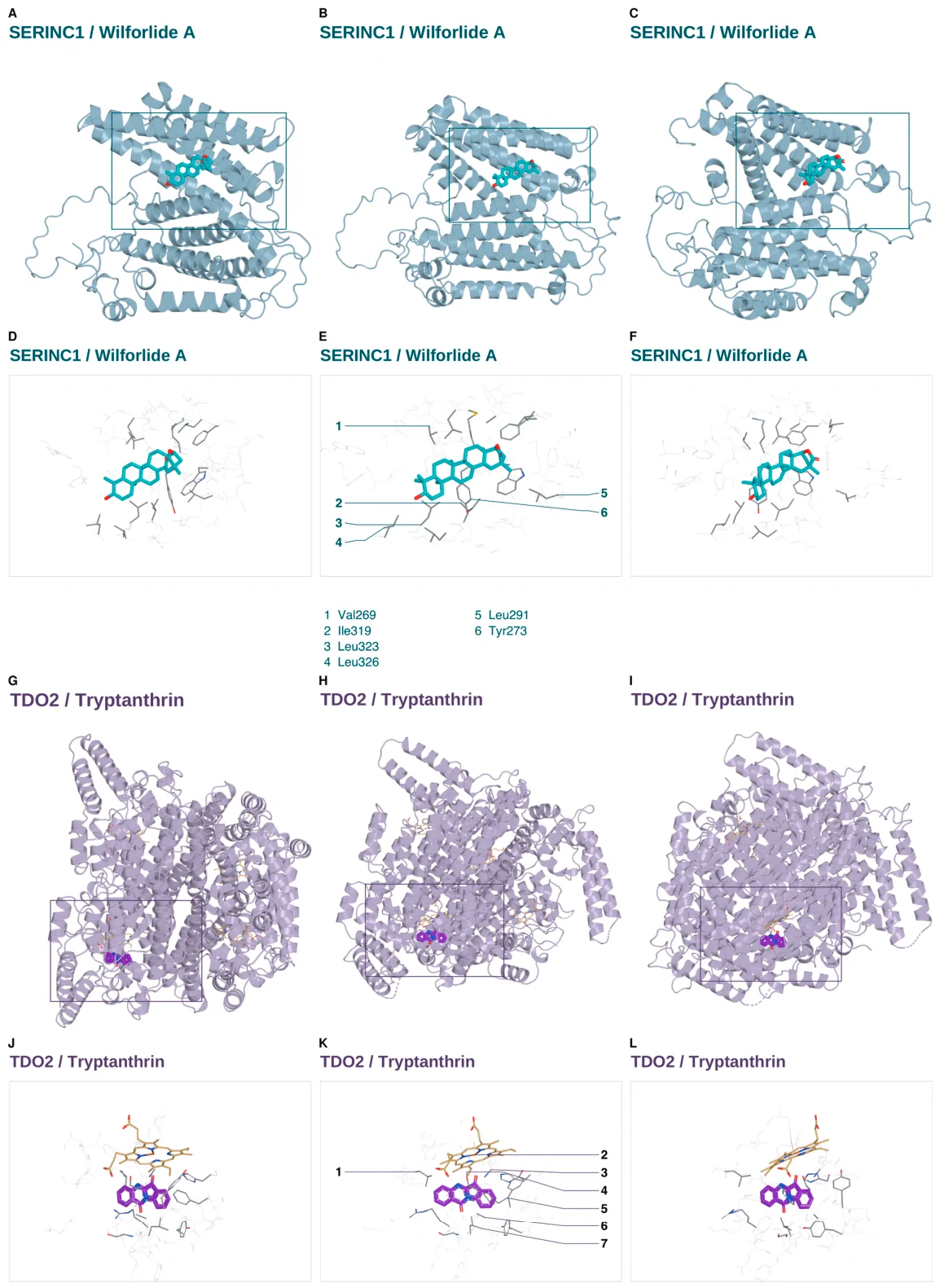
Exploratory molecular docking of SERINC1 and TDO2. (**A**–**F**) SERINC1 (AlphaFold AF-Q9NRX5-F1) docked with Wilforlide A: overall and pocket views at three camera angles (−35°, 0°, and +35°); the selected pose (median best-pose Vina score −10.319 kcal/mol, all three poses passing quality control) contacts six key residues within 4.5 Å (Val269, Ile319, Leu323, Leu326, Leu291, and Tyr273; closest contact ~2.732 Å). (**G**–**L**) TDO2 (tetrameric PDB 8VUG) docked with Tryptanthrin: overall and pocket views at three camera angles; the selected pose (Vina score −9.635 kcal/mol; inter-seed RMSD 0.028–0.122 Å) contacts six key residues and the chain B haem (In panel (**K**), the numbers 1–7 denote Leu336, Gly152, Ser151, Phe72, Ala150, Leu147, and the haem group, respectively). Docking shows ligand–receptor geometry only and does not establish binding affinity, inhibition, or functional effect. Different colors in (**E**,**K**) represent different structural features.

For SERINC1, P2Rank pocket 5 passed the criteria (mean lining-residue pLDDT 92.12). Among six chemical states, Wilforlide A had the lowest median best-pose Vina score (−10.319 kcal/mol), all three poses passing quality control; the selected pose contacted six residues within 4.5 Å (Val269, Ile319, Leu323, Leu326, Leu291, and Tyr273; closest contact ~2.732 Å; Figure 7A–F). The low pLDDT of Leu291 (59.16) leaves the local contact uncertain. Wilforlide A is testable within this scope, but docking does not imply enhanced function. For TDO2, PPN1 redocking achieved superposition-free RMSD 1.710–1.715 Å (threshold 2.0 Å), and all Tryptanthrin best poses passed quality control; the selected pose (median of seeds) scored −9.635 kcal/mol (inter-seed RMSD 0.028–0.122 Å), contacting six residues and the chain B haem (Leu336, Gly152, Ser151, Phe72, Ala150, and Leu147; Figure 7G–L). The three views display the same pose; Vina scores were not compared across receptors, and proximity was not equated with binding, inhibition, or therapy. Missing membrane (SERINC1) and rigid haem/approximate metal (TDO2) further limit interpretation.

### 2.8. Renal Pathology and Ultrastructural Changes in STZ Mice

**Figure 8:**
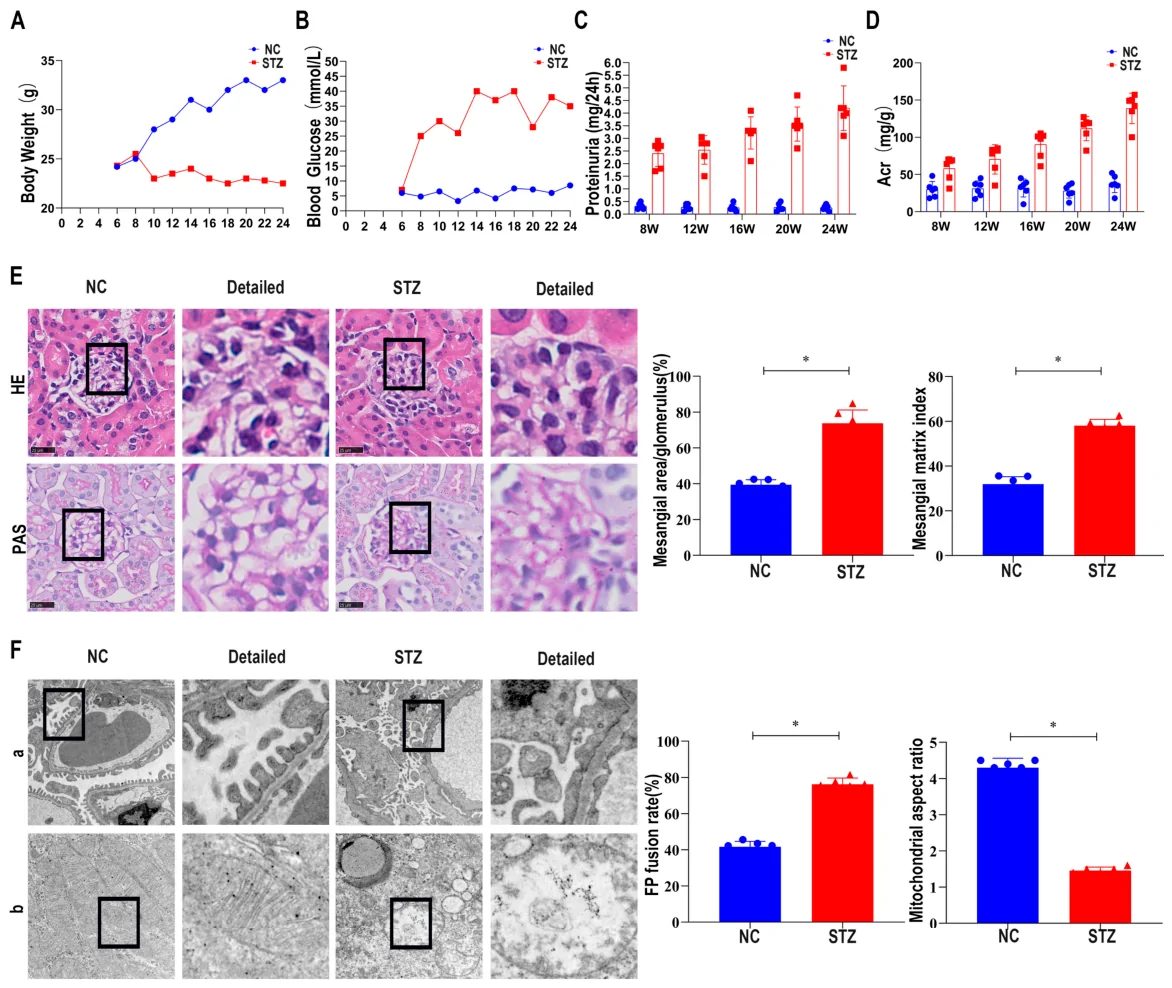
Renal pathology and ultrastructure in control and 24-week STZ mice. (**A**) Body weight; (**B**) blood glucose; (**C**) urinary protein; and (**D**) albumin to creatinine ratio. (**E**) H&E and PAS staining (×400), and scale bars: 25 µm. (**F**) TEM of capillary loops ((**a**), ×12,000) and mitochondria (**b**). NC, non-diabetic control; STZ, streptozotocin-induced diabetes. * *p* < 0.05 versus NC.

Compared with NC, STZ mice showed increased blood glucose and albumin to creatinine ratio and decreased body weight at 24 weeks (Figure 8A–D), increased glomerular matrix (Figure 8E) and foot-process effacement, basement-membrane thickening, and mitochondrial swelling, vacuolation, reduced cristae, and lower matrix density on TEM (Figure 8F).

### 2.9. Expression of SERINC1 and TDO2 in the Mouse Model and Glomerular Staining Distribution

**Figure 9:**
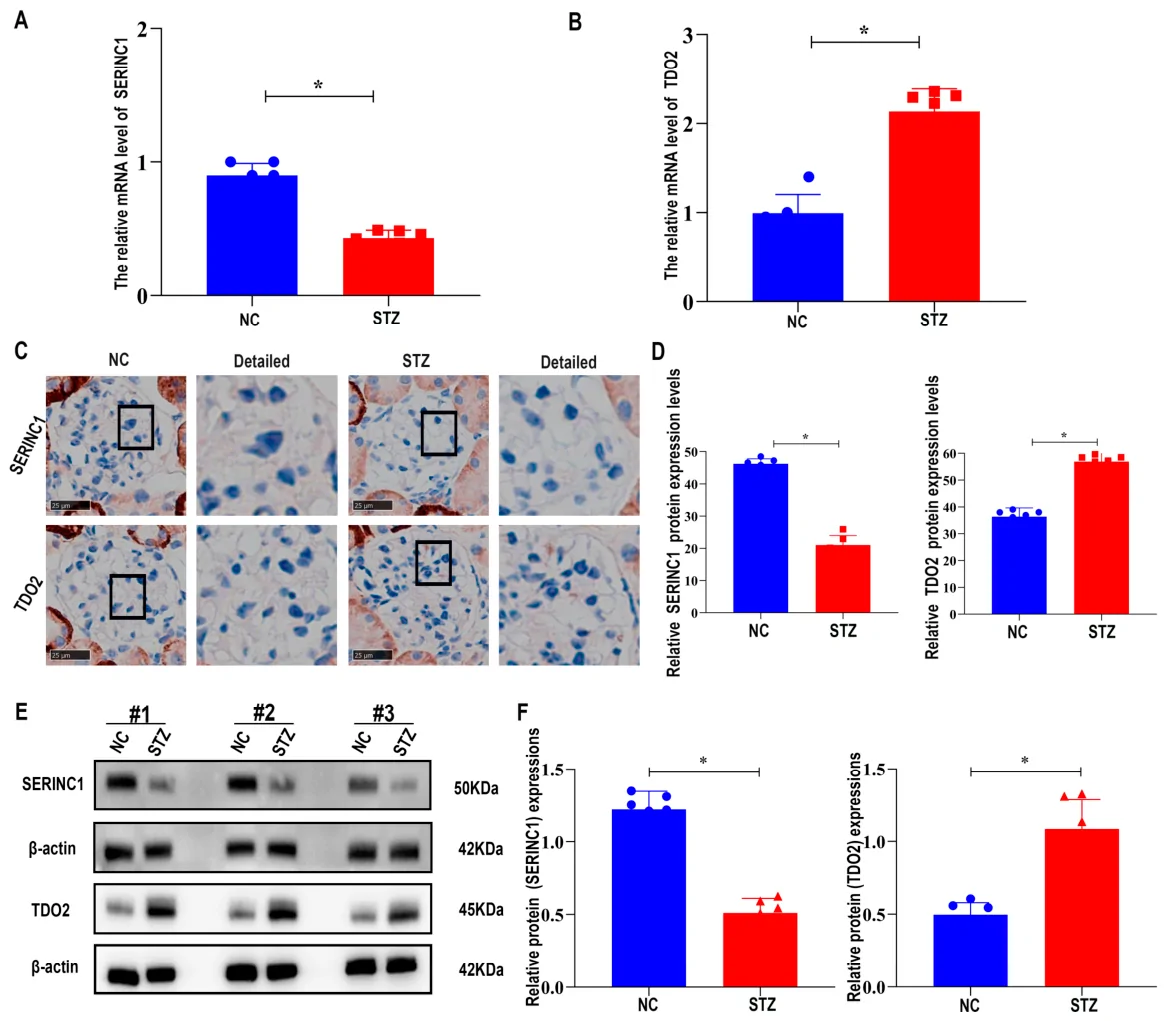
SERINC1 and TDO2 mRNA and protein in mouse kidney. (**A**,**B**) qRT-PCR (SERINC1 decreased, TDO2 increased). (**C**) IHC images; (**D**) IHC quantification. (**E**) Western blots: #1, #2, and #3 denote three independent biological replicate pairs; each pair comprised one normal control (NC) and one STZ-induced diabetic (STZ) mouse that were randomly grouped for imaging. (**F**) ImageJ densitometry normalized to β-actin. * *p* < 0.05 versus NC.

**Figure 10:**
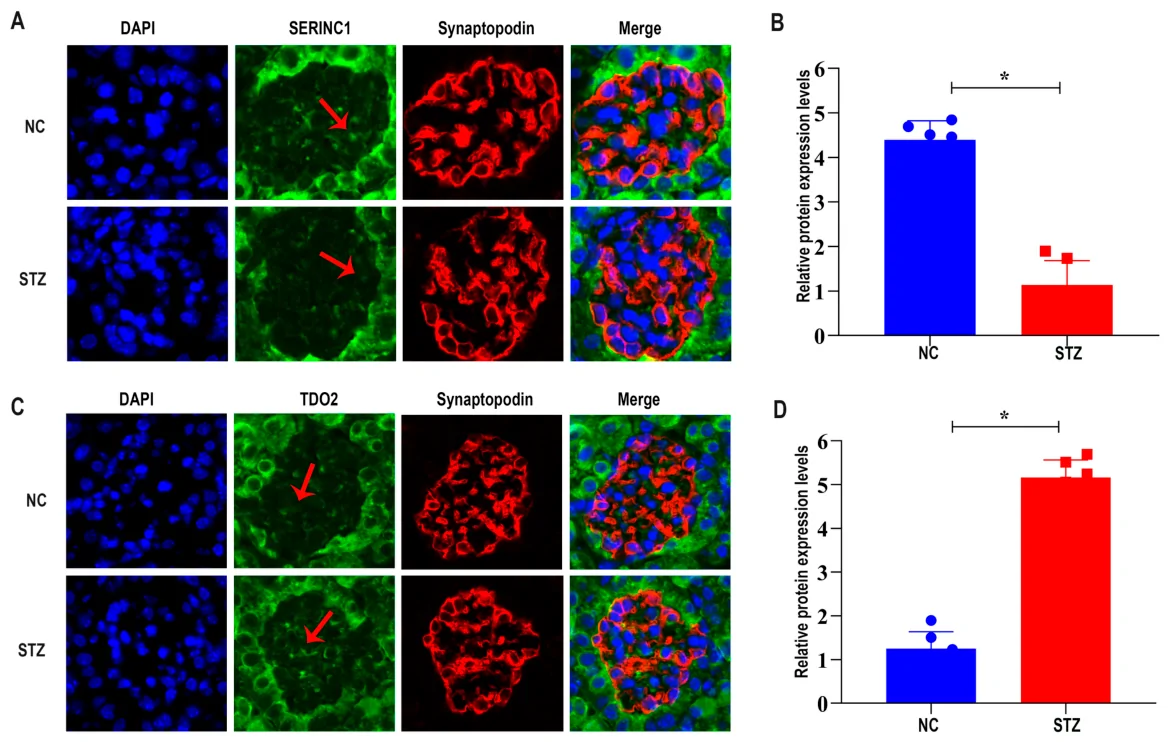
Combined immunofluorescence with Synaptopodin. (**A**,**B**) SERINC1 (green) with Synaptopodin (red) and quantification (reduced in STZ). (**C**,**D**) TDO2 (green) with Synaptopodin (red) and quantification (increased in STZ). Red arrows point to the positive signals of gene expression. * *p* < 0.05 versus NC. Multi-channel signals describe localization, not direct interaction or causality.

qRT-PCR showed lower SERINC1 and higher TDO2 mRNA in STZ mice (Figure 9A,B), with protein differences in the same directions by immunohistochemistry and Western blotting (Figure 9C–F). Immunofluorescence with Synaptopodin showed reduced membrane-associated SERINC1 and increased cytoplasmic-like TDO2 (Figure 10A–D). The SERINC1 reduction matched the discovery cohort but not GSE96804, whereas TDO2 matched both cohorts; expression and staining differences alone do not establish causation.

## 3. Discussion

The behavior of the two genes across different data levels constitutes the main finding of this study. TDO2 was increased in both glomerular cohorts and in STZ mice, whereas the reduction in SERINC1 was seen in the historical discovery cohort and in the mouse model but not in GSE96804. Figure 1 aligns candidate origin with cross-cohort evidence, clarifying how historical inclusion, current screening, and subsequent validation relate. Tissue expression, cellular distribution, and animal observations delimit the research value of the two genes, but no evidence currently places them on a shared pathogenic axis.

Interpreting TDO2 requires separating expression, enzyme activity, and flux. TDO2 and IDO catalyze the initial reactions of the kynurenine pathway, but a transcript increase does not prove increased pathway flux. Tsai et al., combining single-cell, diabetic animal, and clinical data, reported changes in immune cells and in the kynurenine pathway; the proximal tubule-related enzymes in that work involve KYNU and cannot be transferred directly to a podocyte TDO2 role [14]. We did not measure tryptophan depletion, GCN2 stress, accumulation of 3-hydroxykynurenine or quinolinic acid, or NAD+ flux; the present results support measuring these processes rather than predefining downstream mechanisms from expression changes [8]. Erdem et al. reported cross-sectional discrimination of DKD by kynurenic acid, IDO, and 3-hydroxykynurenine in 164 individuals, with a maximum AUC of 0.951; that value is a performance estimate for circulating metabolites and a study of IDO, not for the TDO2 gene [13,15]. Our tissue-level estimates (TDO2 AUC 0.660 in GSE96804; two-gene ANN 0.609) are not yet sufficient to support clinical prediction. Intervention studies also require disease context: M4112 did not persistently lower plasma kynurenine in a phase I solid-tumor study that was discontinued for insufficient pharmacodynamic effect [16], and in the phase III ECHO-301/KEYNOTE-252 trial, epacadostat plus pembrolizumab did not improve progression-free or overall survival [17]. Oncology results cannot directly predict therapeutic effects in DKD. The kynurenine to tryptophan ratio (KTR) is a measurable follow-up marker but is not TDO2-specific.

The membrane lipid-related hypothesis for SERINC1 needs cell-specific verification [18]. Early work linked the Serinc family to serine incorporation into membrane lipids and reported rat Serinc1 co-localization with endoplasmic reticulum lipid-synthesis enzymes, whereas later Serinc1 loss-of-function studies found no significant lipid or functional changes in macrophages and lymphocytes [19,20]. PGK1 and LRH-1/PLIN5 studies connected serine metabolism, mitochondrial respiration, podocyte senescence, and lipid droplet turnover with injury, in relevant models, but none validated SERINC1 [21,22]. Our observed reduction with membrane-associated staining generates hypotheses; however, the inconsistent cross-cohort results require simultaneous consideration of disease stage, clinical exposure, and tissue cellular composition.

The single-cell data did not resolve the origin of the two genes in human podocytes. TDO2 showed only 50 non-zero counts in 37,331 cells (35 in the Unresolved class) and failed expression filtering in all eight donor-replicated cell types; low abundance, droplet capture, donor composition, and annotation differences may each contribute and cannot be distinguished here [7,21]. SERINC1 was more widely detected, but donor-level differences were not significant after family-wise BH correction across the 28-test family. Without an independently comparable podocyte population, the mouse co-localization cannot be extrapolated to humans; RNAscope with reliable cell markers or spatial assays of appropriate resolution, and verification of target transcript detection in future single-nucleus data, are indicated [7]. The inconsistency of SERINC1 between bulk cohorts might reflect shifts in cell-population composition rather than cell-intrinsic transcriptional regulation. Donor-level pseudobulk decomposition detected no significant within-cell-type change after family-level correction, a result consistent with a compositional or technical effect, the causal basis of which warrants independent verification.

Historical origins shape the interpretation of the model results. The legacy RFE retained all 61 input variables, and its script implementation is uncertain, so the three-list intersection is not independent concordant evidence from three validated algorithms; DUSP1 from the current six-gene screen belongs to another candidate space and cannot substitute for evaluating the fixed panel [23,24]. Nested cross-validation within the fixed panel did not cover the original discovery process, and the performance decline in GSE96804 indicates sensitivity to distributional shifts in new samples. Colocalization gave no strong support for shared causal variants (H4 approximately 0.86% and 1.03%), but the shared sets covered only about one third of the variants in the original GWAS regions and omitted the GWAS leading variants of both regions [25]. With only 73 kidney-cortex eQTL samples, weak signals, the single-causal-variant assumption, and sensitivity to the priors and scale, a low H4 neither establishes nor excludes a biological association; this analysis did not estimate causal effects, and better-powered data with complete regional coverage are needed. The docking analyses were intended to generate testable hypotheses rather than to screen clinical candidates, and the two ligands were selected for their structural or biological relevance to the corresponding gene. Tryptanthrin, an indoloquinazoline alkaloid, was paired with TDO2 because its indole moiety is structurally analogous to the tryptophan substrate of this enzyme [12]. Six Tripterygium-derived compounds (triptolide, celastrol, triptonide, wilforlide A, wilforlide B, and demethylzeylasteral) were ranked by their median best-pose Vina score, and Wilforlide A, with the lowest score, was selected for display. The structural analysis produced experimentally testable poses for the specified receptors and search boxes. Vina scores are not binding affinities and cannot be ranked across receptors; the predicted SERINC1 structure lacks a membrane environment, and TDO2 was treated with a rigid haem and approximate metal handling [26,27]. Even if binding were shown experimentally, effects on SERINC1 activity, expression, or membrane localization would still require direct tests; functional rescue cannot be inferred from docking.

Future functional studies could prioritize TDO2, whose tissue expression direction is relatively consistent: after the cellular origin has been confirmed, gene intervention, enzyme activity, and metabolite measurements should be combined to test effects on podocyte injury, with rescue experiments for specificity. In viral hepatitis models, Tdo2 deletion led to metabolite compensation without a compensatory rise in IDO1 or IDO2 expression or activity; renal compensation must be measured directly [28]. TDO2 effects should also be separated from IDO1 and other small-molecule targets, and the available evidence does not favor dual inhibition. For SERINC1, whether knockdown or restoration of expression changes membrane lipid composition and injury should be tested first, before discussing the form of intervention. Downstream redox metabolites and NAD+ flux are testable questions, and SGLT2-, GLP-1-, and PPAR-related metabolic interventions cannot substitute for direct functional evidence on the target genes.

Limitations include sample size and analytical dependence. The two glomerular cohorts are small, with incomplete age, sex, renal function, and treatment information; GSE96804 participated in the current genome-wide screening and cannot validate its new candidates; validation standardization used the whole cohort and did not test single-patient deployment; and the single-cell data had only three donors per group, with tissue sampling changing from glomerulus to kidney cortex, so no podocyte-specific comparison or significant fixed-panel pseudobulk result was obtained. The statistical limitations of the pathway and colocalization analyses and the structural approximations of the docking are not removed by expression differences at other levels [29]. Substantial between-cohort heterogeneity (270 DEGs with a significant cohort × disease interaction) further cautions against over-interpreting the pooled estimates; concordance in direction alone does not eliminate concerns about cross-cohort heterogeneity. The animal experiments involved only male STZ mice, and a 24-week observation cannot represent the full course of human DKD; concurrent expression and pathology changes do not establish their temporal order. Fibrosis markers (collagen I/IV, fibronectin, and α-smooth muscle actin) were not measured, and the single 24-week endpoint cannot establish whether the expression changes precede, coincide with, or follow renal injury; a time-course design with functional anchoring is required to distinguish driver from consequence. Per-animal expression–phenotype correspondence, podocyte-specific single-gene intervention, and rescue experiments remain necessary, and the source of experimental samples, the actual replicate numbers, and assay specificity must be documented in full to judge whether the population and model results address the same question [30]. Reproducibility should be tested in independent cohorts with standardized clinical information, and the mechanistic hypotheses assessed with kynurenine- and serine-related metabolite measurements.

The available evidence supports distinct validation priorities: for TDO2, confirm the cellular origin and then test function; for SERINC1, additionally explain the context dependence of the expression differences. Retaining the negative, non-estimable, and inconsistent results for both genes helps to define the specific questions of the next experiments, and direct evidence is still needed on whether the two genes regulate each other and jointly affect DKD progression.

## 4. Materials and Methods

### 4.1. Glomerular Transcriptome Preprocessing, Differential Expression, and Co-Expression Analysis

Human glomerular transcriptome cohorts GSE30528 (13 control, 9 DKD) and GSE96804 (20 control, 41 DKD) were downloaded from Gene Expression Omnibus (GEO, https://www.ncbi.nlm.nih.gov/geo/, accessed on 14 July 2026) (83 samples). Each cohort was quantile-normalized separately and merged by common gene symbols (11,406 genes); probe-level expression was collapsed to the gene level by selecting the probe with the highest mean expression. ComBat (sva v3.50.0, Bioconductor) corrected cohort/platform differences with disease group protected, and principal component analysis (PCA) checked sample structure before and after correction. The corrected matrix served for visualization, co-expression, and candidate reduction; formal differential testing used the pre-correction matrix. limma v3.58.1 (Bioconductor) included batch and disease group with robust empirical Bayes (mean-variance trend); differentially expressed genes (DEGs) were defined as BH-adjusted FDR < 0.05 and |log2FC| > log2(1.5), and cross-cohort directions and the cohort × group interaction were examined. All analyses were performed in R v4.3.1 (R Foundation for Statistical Computing, Vienna, Austria) with Bioconductor 3.18.

Weighted gene co-expression network analysis (WGCNA v1.72-5, R package) used the 1440 genes with SD > 0.5, biweight midcorrelation, a signed network, and signed topological overlap matrix (soft threshold 12, deepSplit 2, and minimum module size 50; modules with eigengene height < 0.30 merged); module-DKD association included disease status and batch. DEGs with concordant cross-cohort direction were intersected with the most disease-associated module. Gene Ontology (GO), KEGG, and Disease Ontology enrichment (clusterProfiler v4.10.0 and DOSE v3.28.2, Bioconductor) used the WGCNA input genes as primary background, with sensitivity analyses on all tested genes and the target module; corrections were separate, with all testable GO terms forming one BH family per background (2431 tests). STRING v12.0 (https://string-db.org/, accessed on 14 July 2026) networks used confidence 0.700 and 0.400 with isolated nodes retained; topological ranking combined Degree, MNC, MCC, and EPC percentiles. The 26-gene intersection was reduced to six topology-derived candidates by the topological percentile ranking.

The six topology-derived genes entered LASSO (glmnet v4.1-8), linear SVM-RFE (caret v6.0-94), and a probabilistic random forest (randomForest v4.7-1.1). LASSO selected by cross-validated λ and non-zero coefficients, SVM-RFE by recursive elimination with cross-validation, and the random forest by permutation importance within training splits. All applied the one-SE rule (the simplest model whose cross-validation error is within one standard error of the minimum), with nested five-fold outer/inner cross-validation repeated 20 times (frequencies over 100 outer models). These models describe the reduction context only: SERINC1 and TDO2 did not enter and received no scores. Because upstream screening and ComBat used all 83 samples, the metrics reflect conditional internal performance and are not a fully independent estimate of the entire feature-selection pipeline.

SERINC1 and TDO2 stem from the historically fixed panel, reappraised via the historical WGCNA green module, LASSO list, and the list originally labelled SVM-RFE. The historical LASSO was recomputed from GSE30528 (binomial, α = 1, standardized inputs, 10-fold cross-validation, λ by binomial deviance; Figure 1A,B). The legacy RFE script used methods = “svmRadial” instead of method = “svmRadial”, converted the outcome to numeric, and did not retain its fitted object; Figure 1C restores the historical curve from the original PDF as RFE*, not a confirmed SVM re-run. The frozen-list intersection documents provenance only (Figure 1D). Neither gene met the DEG threshold or entered the candidate set; both were retained throughout, without modifying quality control, annotation, or screening thresholds.

### 4.2. Cohort Evaluation of the Fixed Two-Gene Panel and Cross-Cohort Transfer of the Predictive Model

The fixed panel was developed in GSE30528 (historical reproduction) and evaluated at the candidate level and as the locked model in GSE96804. GSE30528 ROC analysis is reuse of discovery data; GSE96804 did not contribute to the historical intersection but participated in the current genome-wide analysis, so it cannot validate the new candidates. Within-cohort differences were estimated with limma (Holm-adjusted) and reported as Hedges’ g with 95% CIs; candidate-level confirmation required the historical direction and a two-sided Holm-adjusted *p* < 0.05. The direction of association was fixed a priori (low SERINC1, high TDO2 = DKD); AUC CIs used DeLong (pROC v1.18.5). Figure 1E–H,J,K show the discovery-reuse ROCs, the GSE96804 ROCs/precision-recall, and the cross-cohort AUCs/effect sizes. Average precision (AP) was compared with the cohort DKD proportion.

The two genes were z-standardized within cohort and entered a Firth penalized logistic regression with mutual adjustment; ORs per 1 SD came from the profile likelihood (Holm-adjusted). Age, sex, and renal function were omitted (no consistent covariates) [31]. The ANN (neuralnet v1.44.2) had two input nodes, one hidden and one output node (sigmoid, cross-entropy). Development used 20× outer five-fold cross-validation with inner four-fold weight-decay selection (0.0001, 0.001, 0.01, 0.1, and 1) by Brier score; standardization used training folds only, with multiple random initializations. Final tuning used 20× four-fold and 50 initializations, and the threshold was locked from the Youden index derived exclusively from the out-of-fold predictions. An L2-penalized logistic model (glmnet v4.1-8) was fit on the same splits. Metrics: AUC, AP, Brier, sensitivity, specificity, and calibration intercept/slope. Figure 1I shows the locked ANN response surface within the development rectangle (grey outside); contours at P(DKD) 0.25/0.50/0.75 aid interpretation only.

GSE96804 was z-standardized with its cohort mean and SD (no outcome labels), assessing cohort-level transferability rather than single-patient deployment. Cross-cohort AP and calibration uncertainty used 1000 stratified bootstrap resamples; empirical quantiles describe resampling stability, not independent-sample CIs.

### 4.3. Single-Cell RNA Sequencing Analysis

GSE209781 provided human kidney-cortex scRNA-seq (10× Genomics) from three control (NM01–NM03) and three DKD (DKD01–DKD03) donors; analysis started from submitter-provided UMI matrices aligned to GRCh38. Cell-containing droplets were identified per library with emptyDrops from DropletUtils v1.22.0 (lower = 100; 10,000 iterations; FDR ≤ 0.01). Quality control required ≥300 genes, ≥500 UMIs, and ≤25% mitochondrial as well as ≤10% hemoglobin transcripts, followed by within-sample upper outlier thresholds (median + 3 × MAD; log1p for counts, original scale for fractions; caps 8000 genes, 100,000 UMIs). Doublets were removed with scDblFinder v1.16.0.

Cells were normalized in Seurat v5.0.1 (LogNormalize, scale factor 10,000; 2000 variable genes), scaling regressed out the mitochondrial fraction, and 30 PCs were computed. Harmony v0.1.1 integrated the PCs with donor ID as a covariate, and the first 20 dimensions built the neighbor graph and UMAP. Clustering used a resolution of 0.4 (0.2–1.2 compared). Cell types were annotated from canonical markers and co-expression; SERINC1 and TDO2 did not participate. SingleR v2.4.1, using the Human Primary Cell Atlas (HPCA) reference via the celldex package, provided auxiliary scores; cells with insufficient evidence were Unresolved. All analyses were performed in R v4.3.1 (R Foundation for Statistical Computing, Vienna, Austria) with Bioconductor 3.18.

The main figure shows two representative markers per cell type from the largest cluster. Because donor ID is nested within disease group, post-Harmony mixing does not prove disease-signal preservation; integrated coordinates served representation/clustering only, and differential testing used raw counts.

### 4.4. Single-Cell Visualization and Pseudobulk Differential Analysis

Distribution of SERINC1 and TDO2 was shown via feature plots, expression-distribution plots, and detection-proportion plots, grouped by condition, retaining zero-count cells without imputation [32]. Groups shared coordinate ranges, and each gene’s two plots shared a color scale capped at the 99th percentile of combined non-zero expression (display only). Raw counts were aggregated by donor and cell type into pseudobulk matrices; inference included only cell types with ≥20 cells per donor in all six donors (Unresolved excluded).

Matrices were TMM-normalized, filtered with filterByExpr, and modelled in edgeR v4.0.2 (Bioconductor) (robust quasi-likelihood negative binomial) with disease group to estimate log2FC. The family comprised 2 genes × 14 cell types (28 hypotheses), with non-estimable items still counted in the BH family; distributions are shown as log2(CPM + 0.1). Items with insufficient cells or failed filtering were marked as not estimable; cell-level proportions were descriptive only.

### 4.5. Transcriptome Association, Pathway Enrichment, Candidate Regulatory Network, and Colocalization

Within each cohort’s DKD samples (9 and 41), linear models regressed continuous expression of each target gene on the remaining 11,405 genes, ranked by limma empirical Bayes (mean-variance trend; target excluded). A local KEGG collection (KEGG, https://www.genome.jp/kegg/, accessed on 14 July 2026) retained 164 pathways (15–500 genes); CAMERA (inter-gene correlation) was primary, with the 328 tests per cohort BH-corrected together, and fgseaMultilevel supplied ranking curves, NES, and sensitivity [33,34]. Figure 6C shows the peroxisomal leading-edge intersection as raw moderated t (alphabetical, shared symmetric scale, and no row scaling).

The candidate network integrated local TargetScan v8.0 (https://www.targetscan.org/vert_80/), miRDB v6.0 (https://mirdb.org/), miRanda (microRNA.org, release August 2010), and miRTarBase v10.0 (https://mirtarbase.cuhk.edu.cn/), all accessed on 14 July 2026 (exact gene symbol and miRNA identifier; distinct 5p/3p retained; and within-source duplicates not independent). After joining spongeScan (https://academic.oup.com/nar/article/44/W1/W176/2499387, accessed on 14 July 2026), only lncRNAs approved by the HUGO Gene Nomenclature Committee (HGNC, https://www.genenames.org/, accessed on 14 July 2026) entered. These simplified local databases lack verifiable versions and context, so the network displays candidate relationships only.

Because the planned eQTLGen/OpenGWAS data were unavailable, a supplementary colocalization analysis was performed (labelled as such; not a Mendelian randomization result) using GTEx kidney-cortex eQTLs (eQTL Catalogue, datasets QTS000015 and QTD000261, https://www.ebi.ac.uk/eqtl/, accessed on 14 July 2026; 73 individuals) and FinnGen R12 DM_NEPHROPATHY (https://www.finngen.fi/, accessed on 14 July 2026; 5579 cases, 90,951 diabetic controls) [35]. The FinnGen phenotype is a case-control trait, and the case fraction and per-variant effect estimates and standard errors were provided to coloc accordingly. For each gene, published cis-eQTL variants were intersected with the GWAS region and matched exactly on GRCh38 position and alleles (alternative allele as effect allele; indels retained; and no *p*-value filtering). Colocalization used coloc 5.2.3 approximate Bayes factors (one causal variant per region/trait), with priors p1 = p2 = 1 × 10^−4^, p12 = 1 × 10^−5^, sdY = 1; sensitivity varied sdY (0.5, 2, estimated) and p12 (1 × 10^−6^ to 1 × 10^−4^). Posteriors H0–H4 and shared-variant coverage of the original regions/leading variants were examined.

### 4.6. Local Molecular Docking and Multi-View Structural Display

Exploratory docking paired SERINC1 with Wilforlide A (PubChem CID 158477) and TDO2 with Tryptanthrin (CID 73549), both retrieved from PubChem (https://pubchem.ncbi.nlm.nih.gov/, accessed on 14 July 2026). For SERINC1, pockets were predicted by P2Rank 2.5.1 on the AlphaFold structure AF-Q9NRX5-F1-model_v6 (AlphaFold Protein Structure Database, https://alphafold.ebi.ac.uk/, accessed on 14 July 2026); selection required mean lining-residue pLDDT ≥ 80 and ≥80% residues ≥ 70, and the highest-ranked qualifying pocket was docked (26 × 26 × 26 Å box centered at 4.4213, 9.1055, −2.7998 Å). Six Tripterygium-derived compounds (triptolide, celastrol, triptonide, wilforlide A, wilforlide B, and demethylzeylasteral) were ranked by the median best-pose score over three seeds; the top candidate had to pass pose quality control, without substitution. For TDO2, the tetrameric PDB 8VUG (RCSB Protein Data Bank, https://www.rcsb.org/, accessed on 14 July 2026; four chains, four haems) was used; water, the co-crystal ligand and other hetero-ligands were removed, missing fragments were not remodeled, and the box (26 × 26 × 26 Å) was centered on the PPN1 heavy-atom centroid at chain B residue 403 (−12.96496, 19.56288, and 14.61216 Å).

TDO2 ligands were generated by RDKit v2023.09.3 ETKDGv3 (10 conformers) with MMFF94s optimization and prepared with Meeko v0.5.0 (PPN1 +1 charge, protonated piperidine; Tryptanthrin neutral diketone). AutoDock Vina 1.2.7 (exhaustiveness 32, 20 poses, 5 kcal/mol range, and seeds 20260908–20260910) docked TDO2 after redocking validation (≥2 seeds’ best pose, superposition-free symmetry-corrected heavy-atom RMSD ≤ 2.0 Å). Pose quality control checked chemical identity, geometry, box boundaries, clashes, and pocket contacts (plus missing-fragment distance and cross-seed consistency for TDO2).

The displayed pose was the quality-controlled one whose top score matched the median of the three seeds (4.5 Å neighborhood). PyMOL v2.5.0 adjusted only camera angles (−35°, 0°, and +35°; overall and pocket views, four-by-three grid). SERINC1 lacks a membrane environment and TDO2 used a rigid haem with approximate metal handling; leader lines label residues only.

### 4.7. Animal Model

Six-week-old male C57BL/6J mice (20–25 g) were purchased from Cyagen Biosciences (Suzhou, China) and housed at 22 ± 2 °C under a 12 h light/dark cycle with free access to standard chow and water. After one week of acclimatization, mice were randomly allocated to the normal control (NC) and STZ-induced diabetic (STZ) groups using a random number table (six mice per group); the sample size followed established practice in comparable studies of diabetic kidney disease. Before treatment, baseline body weight and fasting blood glucose were measured using a portable glucometer on tail-vein blood (Accu-Chek Performa, Roche Diagnostics, Mannheim, Germany). Diabetes was induced by five consecutive daily intraperitoneal injections of streptozotocin (STZ, 50 mg/kg, Sigma-Aldrich, St. Louis, MO, USA, dissolved in citrate buffer, pH 4.5) after a 4 h fast; control mice received an equal volume of citrate buffer. Diabetes was confirmed by sustained blood glucose > 16.7 mmol/L one week after the final injection. Body weight and blood glucose were recorded every two weeks thereafter, and 24 h urine was collected every four weeks using metabolic cages (Tecniplast S.p.A., Buguggiate, Italy). Urinary albumin was quantified with a mouse albumin ELISA kit (Abcam, Cambridge, UK), urinary creatinine with an enzymatic creatinine assay kit (Sigma-Aldrich, St. Louis, MO, USA), and the urine albumin to creatinine ratio (ACR) was calculated as albumin (μg/mL)/creatinine (mg/mL); total urinary protein was determined by the BCA Protein Assay Kit (Pierce, Thermo Fisher Scientific, Waltham, MA, USA). At 24 weeks, mice were anaesthetized with pentobarbital sodium (60 mg/kg, intraperitoneal; Sigma-Aldrich, St. Louis, MO, USA) and euthanized by cervical dislocation, and kidneys were collected for histology, immunostaining, and molecular assays. No animals died or were excluded during the experiment, and histopathological assessment was performed by an investigator blinded to group allocation. All procedures were approved by the Ethics Committee of Renmin Hospital of Wuhan University (approval no. WDRM 20230306; approved on 16 March 2023) and complied with the ARRIVE 2.0 guidelines.

### 4.8. Immunohistochemistry

Paraffin kidney sections (4 µm) were dewaxed, rehydrated, and microwave-retrieved in citrate buffer (pH 6.0). Endogenous peroxidase (3% hydrogen peroxide) and non-specific binding (5% normal goat serum) were blocked, and sections were incubated overnight at 4 °C with antibodies against SERINC1 (TDE2, 1:200, Cloud-Clone Corp., Wuhan, China) and TDO2 (HYPTRP, 1:200, ImmunoWay Biotechnology, San Jose, CA, USA). After secondary antibody, DAB, and hematoxylin, glomerular staining was recorded under a light microscope (Olympus BX53, Olympus Corporation, Tokyo, Japan); cellular attribution was judged together with immunofluorescence. For semi-quantitative analysis, ten fields containing glomeruli were randomly selected per section, and the staining intensity (integrated optical density) was measured with ImageJ (Fiji) v2.16.0/1.54p (NIH, Bethesda, MD, USA); the mean value of the ten fields was used as a single data point per animal.

### 4.9. Immunofluorescence

After dewaxing, heat retrieval, and blocking, sections were incubated overnight at 4 °C with anti-SERINC1 (1:100; Cloud-Clone Corp., Wuhan, China), anti-TDO2 (1:100; ImmunoWay Biotechnology, San Jose, CA, USA), and anti-Synaptopodin (polyclonal; Progen Biotechnik, Heidelberg, Germany; 1:100), followed by fluorophore-conjugated goat anti-rabbit and goat anti-mouse secondary antibodies (1:500; Thermo Fisher Scientific, Waltham, MA, USA) for 60 min in the dark, followed by DAPI staining and confocal microscope (Leica TCS SP8, Leica Microsystems GmbH, Wetzlar, Germany) multi-channel scanning. With Synaptopodin, glomerular distribution and intensity were examined for spatial localization only. For quantification, ten fields were randomly selected per section and the fluorescence intensity was measured with ImageJ (Fiji); the mean value was used as a single data point per animal.

### 4.10. Haematoxylin and Eosin (H&E) and Periodic Acid–Schiff Staining

Kidney tissue was fixed in 4% paraformaldehyde, paraffin-embedded, and cut at 4 µm. Sections were stained with hematoxylin (5 min) and eosin (2 min) or PAS (0.5% periodic acid 10 min, Schiff reagent 20 min, hematoxylin) and examined at ×400 for glomerular matrix and morphology.

### 4.11. Transmission Electron Microscopy

Renal cortex was fixed in 2.5% glutaraldehyde (Electron Microscopy Sciences, Hatfield, PA, USA; 0.1 M phosphate buffer, pH 7.4, 4 °C, 2 h), post-fixed in 1% osmium tetroxide (Electron Microscopy Sciences, Hatfield, PA, USA; 1 h), dehydrated, embedded in epoxy resin, and sectioned at 70 nm on an ultramicrotome (Leica EM UC7, Leica Microsystems GmbH, Vienna, Austria). Sections stained with uranyl acetate and lead citrate were examined by transmission electron microscopy (Hitachi HT7800, Hitachi High-Tech Corporation, Tokyo, Japan) for the basement membrane, foot processes, and mitochondria.

### 4.12. Western Blotting

Glomeruli were isolated from NC and STZ mouse kidneys by magnetic bead perfusion (Dynabeads M-450 Tosylactivated, Invitrogen, Thermo Fisher Scientific, Waltham, MA, USA, on a DynaMag-2 magnet) as previously described [36], and lysed in RIPA buffer containing protease and phosphatase inhibitors. Protein concentration was determined using a BCA assay kit. Equal amounts of protein (30 μg per lane) were separated by SDS-PAGE (Mini-PROTEAN Tetra, Bio-Rad Laboratories, Hercules, CA, USA) and transferred to PVDF membranes (Trans-Blot Turbo, Bio-Rad Laboratories, Hercules, CA, USA), which were blocked with 5% non-fat milk in TBST for 1 h at room temperature. Membranes were incubated overnight at 4 °C with anti-TDO2 (1:500; Biorbyt, Cambridge, UK), anti-SERINC1 (1:500; Biorbyt, Cambridge, UK), and anti-β-actin (1:10,000; MedChemExpress, Monmouth Junction, NJ, USA), followed by HRP-conjugated goat anti-rabbit and goat anti-mouse secondary antibodies (1:500; Thermo Fisher Scientific, Waltham, MA, USA) for 1 h and enhanced chemiluminescence (ECL) substrate (Pierce, Thermo Fisher Scientific, Waltham, MA, USA) detection using a chemiluminescence imaging system (Tanon 5200 Multi, Tanon Science & Technology, Shanghai, China). Band intensities were quantified with ImageJ and normalized to β-actin. The expected molecular weights were approximately 51 kDa (SERINC1), 48 kDa (TDO2), and 42 kDa (β-actin).

### 4.13. Quantitative Real-Time PCR

Total RNA was extracted from isolated glomeruli using TRIzol reagent (Invitrogen, Thermo Fisher Scientific, Waltham, MA, USA) and reverse-transcribed into cDNA (PrimeScript RT reagent kit, Takara Bio Inc., Kusatsu, Japan), and qRT-PCR was performed with SYBR Green (TB Green Premix Ex Taq, Takara Bio Inc., Kusatsu, Japan) on a QuantStudio 5 Real-Time PCR System (Applied Biosystems, Thermo Fisher Scientific, Waltham, MA, USA). Primer sequences were as follows: TDO2 F GCTTTGGACTTCAATGACTTCAG, R GCCGGAACTGTAGACTCTGG; SERINC1 F CTGTTCAGTGGTGGCATCCTCA, R CCTGACTGTTGTTGGAGGTACG; and β-actin F CCAGCCTTCCTTCTTGGGTATG, R GCCGGACTCATCGTACTCC. β-actin served as the internal reference, and relative expression was calculated using the 2^−ΔΔCt^ method. Each sample was assayed in triplicate (technical replicates), with six independent mice per group serving as biological replicates; technical replicates were averaged before statistical testing. Primer specificity was confirmed via melting curve analysis, and the amplification efficiency of each primer pair was validated by standard curve analysis.

### 4.14. Statistical Analysis

Bioinformatic testing units, correction families, and thresholds are defined in the corresponding method sections; nominal and adjusted *p* values are reported separately. Animal data are presented as mean ± SD. Comparisons between the two groups (NC vs. STZ) were performed using two-tailed unpaired Student’s t-test for body weight, blood glucose, urine parameters, qRT-PCR, Western blot densitometry, and immunohistochemical/immunofluorescence quantification. Statistical analyses were performed in IBM SPSS Statistics 22.0 (IBM Corp., Armonk, NY, USA), with *p* < 0.05 considered significant. Six independent mice per group served as biological replicates; technical replicates were averaged before testing and were not treated as independent observations.

## 5. Conclusions

TDO2 was increased in two glomerular transcriptome cohorts and was consistent with its expression direction in STZ mice, whereas the reduction in SERINC1 was supported only in some study contexts. The reappraisal of the historical candidates clarified the origin of the fixed panel and its methodological limitations, and the single-cell analysis added distributional information while exposing the low detection rate of TDO2 and the paucity of podocyte evidence. Both genes remain suitable candidates for further study, but their clinical diagnostic use and a shared pathogenic mechanism have not been established.

## Data Availability

Public datasets analyzed in this study are available from GEO (GSE30528, GSE96804, and GSE209781), the eQTL Catalogue (GTEx kidney-cortex eQTL, QTS000015/QTD000261), and FinnGen R12 DM_NEPHROPATHY. To support reproducibility, the derived resources are deposited in Zenodo (DOI to be assigned at acceptance) and available from the corresponding author on reasonable request, including the historical frozen gene lists (WGCNA green module, 183 genes; LASSO, 10; and legacy SVM-RFE, 61), the processed matrices (11,406 genes; 37,331 single cells; and donor-by-cell-type pseudobulk), the single-cell cell-type assignments and marker panels, the locked model specifications (2–1–1 ANN, weight decay 0.1, and threshold 0.6533; coloc priors p1 = p2 = 1 × 10^−4^, p12 = 1 × 10^−5^), and the analysis scripts (limma, WGCNA, Seurat, edgeR, CAMERA/fgsea, coloc 5.2.3, and AutoDock Vina 1.2.7). The locally compiled regulatory-database records (TargetScan, miRDB, miRanda, and miRTarBase), which lack verifiable version identifiers, are not redistributed.

## Abbreviations

The following abbreviations are used in this manuscript:

DKD	Diabetic Kidney Disease
ACR	Albumin to Creatinine Ratio
ANN	Artificial Neural Network
AP	Average Precision
AUC	Area Under the Curve
CI	Confidence Interval
DEG	Differentially Expressed Gene
FDR	False Discovery Rate
GPI	Glycosylphosphatidylinositol
KTR	Kynurenine to Tryptophan Ratio
GEO	Gene Expression Omnibus
GO	Gene Ontology
KEGG	Kyoto Encyclopedia of Genes and Genomes
STZ	Streptozotocin
IHC	Immunohistochemistry
IF	Immunofluorescence
H&E	Hematoxylin and Eosin
TEM	Transmission Electron Microscopy
ECL	Enhanced Chemiluminescence
NES	Normalized Enrichment Score
OR	Odds Ratio
PCA	Principal Component Analysis
scRNA-seq	Single-Cell RNA Sequencing
qRT-PCR	Quantitative Real-Time PCR
UMAP	Uniform Manifold Approximation and Projection
WGCNA	Weighted Gene Co-Expression Network Analysis
